# Assessment of Chronic Illness Care with the German version of the ACIC in different primary care settings in Switzerland

**DOI:** 10.1186/1477-7525-8-122

**Published:** 2010-10-27

**Authors:** Claudia Steurer-Stey, Anja Frei, Gabriela Schmid-Mohler, Sibylle Malcolm-Kohler, Marco Zoller, Thomas Rosemann

**Affiliations:** 1 Department of General Practice and Health Services Research, University of Zurich, University Hospital of Zurich, Sonneggstrasse 6, CH-8091 Zürich, Switzerland; 2 Department of Internal Medicine, University Hospital of Zurich Rämistrasse, 100, CH-8091 Zürich, Switzerland; 3 Division of Nephrology, University Hospital of Zurich Rämistrasse, 100, CH-8091 Zürich, Switzerland

## Abstract

**Background:**

In Switzerland the extent to which patients with chronic illnesses receive care congruent with the Chronic Care Model (CCM) is unknown.

**Methods:**

According to guidelines we translated the Assessment of Chronic Illness Care (ACIC) into German (G-ACIC). We tested the instrument in different primary care settings and compared subscales with the original testing.

**Results:**

Difficulties encountered during the translation process consisted in the difference of health care settings in Switzerland and USA. However initial testing showed the G-ACIC to be a suitable instrument. The average ACIC subscale scores in Swiss managed care (MC)-, group (GP)- and single handed practices (SP) were higher for MC practices than for group- and single handed practices: *Organization of the healthcare delivery system*: MC mean (m) = 6.80 (SD 1.55), GP m = 5.42 (SD 0.99), SP m = 4.60 (SD 2.07); *community linkages*: MC m = 4.19 (SD 1.47), GP m = 4.83 (SD 1.81), SP m = 3.10 (SD 2.12); *self-management support*: MC m = 4.96 (SD 1.13), GP m = 4.73 (SD 1.40), SP m = 4.43 (SD 1.34); *decision support*: MC m = 4.75 (SD 1.06); GP m = 4.20 (SD 0.87), SP m = 3.25 (SD 1.59); *delivery system design*: MC m = 5.98 (SD 1.61), GP m = 5.05 (SD 2.05), SP m = 3.86 (SD 1.51) and *clinical information systems*: MC m = 4.34 (SD = 2.49), GP m = 2.06 (SD 1.35), SP m = 3.20 (SD 1.57).

**Conclusions:**

The G-ACIC is applicable and useful for comparing different health care settings in German speaking countries. Managed care organizations seem to implement the different components of the CCM in a greater extend than group and single handed practices. However, much room exists for further improvement.

## Introduction

The Assessment of Chronic Illness Care (ACIC) questionnaire was developed to assess if provided care is in alignment with the Chronic Care Model (CCM) [1]. The ACIC aims at organizational teams to help them to identify areas for improvement for chronic illness care and to evaluate the level and nature of improvements made in their system. It is based on the six areas of system change suggested by the CCM that have been shown to influence quality of care [2]: organization of health care, community linkages, self-management support, decision support, delivery system design and clinical information systems.

In Switzerland the extent to which patients with chronic illnesses receive care congruent with the Chronic Care Model (CCM) is unknown. To drive quality improvement programs, compare different health care settings, and evaluate intervention studies, it is necessary to have practical assessment tools in the country's own language. Although preconditions in different health care systems are different, the shortcomings and gaps in chronic illness care addressed by the six areas in the ACIC show analogy between all types of medical settings and in different countries.

We therefore developed a German translation of the ACIC and tested the instrument in different primary care settings in Switzerland.

## Methods

### Assessment of Chronic Illness Care (ACIC)

The ACIC is based on the specific interventions and concepts within the CCM. It consists of 28 items covering the six areas of the CCM: Organization of the healthcare delivery system (6 items), community linkages (3 items), self-management support (4 items), decision support (4 items), delivery system design (6 items), and clinical information systems (5 items). Responses fall within four descriptive levels D, C, B, A of implementation ranging from D "little or none" to A "fully implemented" intervention. Within each of the four levels, respondents choose one of three ratings of the degree to which that description applies. The result is a 0-11 scale, with categories within this defined as follows: 0-2 (little or no support for chronic illness care); 3-5 (basic or intermediate support for chronic illness care); 6-8 (advanced support); and 9-11 (optimal, or comprehensive, integrated care for chronic illness). Subscale scores for the six areas are derived by summing the response. Bonomi et al showed all six ACIC subscale scores to be responsive to health care quality-improvement efforts [2].

### Translation and cultural adaptation

**Figure 1:**
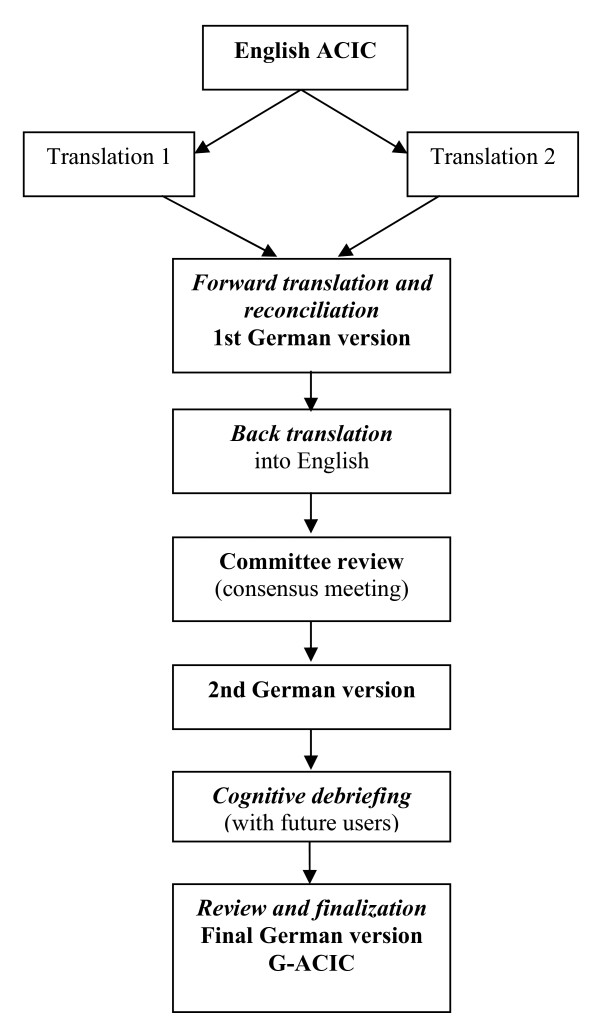
**Translation process**.

After obtaining permission to use and translate the ACIC from the The MacColl Institute for Healthcare Innovation, Group Health Cooperative [3] we followed a translation approach (figure 1) according to the well established guidelines of the "ISPOR Task Force for Translation" and the "World Health Organization's recommendations on the process of translation and adaptation of instruments" in order to achieve the highest possible content validity [4,5], http://www.who.int/substance_abuse/research_tools/en/.

#### Forward translation and reconciliation

Two translators independently translated the English ACIC version into German. Together with a 3^rd ^member of the research group reconciliation was carried out into a single forward translation and first version.

#### Back translation

The back translator who was a native speaker of the original language English and unaware of the original English ACIC translated the 1^st ^ACIC German version back into the source language. In a multidisciplinary consensus meeting where problematic items and translation solutions were discussed a 2^nd ^German version was developed.

#### Cognitive debriefing

The 2^nd ^version was tested for cognitive equivalence and comprehensibility of the translation in a group of health professionals (2 physicians, 2 medical practice assistants, 2 clinical nurse specialists, 1 staff nurse).

#### Review of cognitive debriefing results and finalization

Subsequently, the project team discussed the professionals' comments and issues that caused confusion and adjusted inappropriate items. This process led to the final version of the German-ACIC, the G-ACIC.

### Feasibility testing and validation in different primary care institutions

The final German version was delivered to different primary care settings in Switzerland (two urban managed care organisations (mediX Zürich, SWICA St.Gallen), 11 group practices (6 urban, 5 rural region) and 7 single-handed practices (4 in rural regions and 3 urban) in order to compare subscale scores achieved in these medical organisations with those obtained in the original testing.

Two researchers (AF, CSS) assessed completeness and convergence or dissonance of the G-ACIC instrument data. A sample of three physicians and two medical practice assistants were interviewed individually about acceptance and feasibility.

## Results

The G-ACIC (see additional file 1) comprises 28 items covering the six areas of the Chronic Care Model [2] plus in congruence with the actual version 3.5 of the ACIC [3] six additional items that address how well a practice team or organization integrates the Chronic Care Model elements.

The discrepancies we were faced with and which made some modifications necessary were mainly due to the different health care settings and health care organization in Switzerland. For instance, the health care professionals had difficulties with the items benefits (Part 1: Organization of the Healthcare Delivery System), regional health plans (Part 2: Community Linkages) and planned visits for chronic illness care (Part 3c: Delivery System Design). We discussed the meaning of planned visits with regular planned visits, incorporated patient goals, interactions to support evidence-based care and regular follow-up. Statements varied from: "this is what we do anyway" to "this is not institutionalized in the Swiss health care delivery system". We handled the wide range by using the terms "Folgevisiten/Nachkontrollen" and "Spezifische Visiten für chronische Krankheitsversorgung" and by mentioning the key elements of a planned visit within the four descriptive levels D, C, B, A. Benefits was translated in the meaning of financial support into "Zuschüsse", regional health plans do not exist but health plans in Switzerland are supra regional on a cantonal or national base and national in Germany as well, therefore the translation for regional health plans was adopted to "Kantonale und nationale Gesundheitspläne".

Addressing the fact that the implementation of new tools into daily practice depends largely on practicability and time constraint we asked the health professionals who tested the German translation for comprehensibility to also report on the time needed for the whole process. The time effort ranked between 45 minutes and 20 minutes depending whether the form was filled out in a team consensus approach or individually.

Two managed care (MC) organisations (mediX Zürich, SWICA St.Gallen), 11 group practices (GP) and 7 single-handed practices (SP) completed the G-ACIC for the condition "diabetes". The instrument was completed individually by ten general practitioners and fifteen times together with the medical practice assistants. Table 1 gives an overview of the scores.

**Table 1:** Average ACIC Scores Comparison between different Swiss primary care organizations and average ACIC scores at start of Chronic Care Collaborative tested by Bonomi et al., 2002 (n = 90)

ACIC Subscale Scores												
	***Organization***		***Community linkages***		***Self-management***		***Decision support***		***Delivery system design***		***Information systems***	

***Samples***	***M***	***SD***	***M***	***SD***	***M***	***SD***	***M***	***SD***	***M***	***SD***	***M***	***SD***

Swiss managed Care practices (*n *= 7)	6.80	(1.55)	4.19	(1.47)	4.96	(1.13)	4.75	(1.06)	5.98	(1.61)	4.34	(2.49)

Swiss group practices (*n *= 11)	5.42	(0.99)	4.83	(1.81)	4.73	(1.40)	4.20	(0.87)	5.05	(2.05)	2.06	(1.35)

Swiss single handed practices (*n *= 7)	4.60	(2.07)	3.10	(2.12)	4.43	(1.34)	3.25	(1.59)	3.86	(1.51)	3.20	(1.57)

Overall (combined across collaborative) baseline scores (Bonomi et al., 2002) (*n *= 90)	6.42	(1.82)	5.90	(2.30)	5.41	(2.00)	4.80	(1.99)	5.40	(2.23)	4.36	(2.19)

The average subscale scores ranged from 2.06 to 6.80 indicating limited to reasonably good support for diabetes care. The Swiss managed care (MC) organizations showed better results in most subscales compared to the group practices and single handed practices. The MC scores of decision support, delivery system design, and information systems were comparable to the overall baseline scores measured by Bonomi et al. [2]. The score for health care organization was higher (6.80 vs. 6.42), whereas for the community linkages (4.19 vs. 5.90) and self-management areas (4.96 vs. 5.41) lower scores were obtained (Table 1).

The group practices and single handed practices scored lower in all subscales compared to the baseline scores of the original testing. The group practices showed for five of the six chronic care model elements only basic support and regarding information systems limited support for patients with diabetes. The subscale scores for the single handed practices were below the group practices with the exception of the information systems element (Table 1).

## Discussion

This paper describes the translation of the German version of the ACIC and initial testing in different primary care settings in Switzerland. Our experience shows that the German version is applicable and the results suggest that it is a useful tool to guide quality improvement in chronic illness care in different health care organizations.

During the translation process some modifications considering the different health care systems in USA and Switzerland were necessary. Data from other countries using the ACIC to evaluate the degree of implementation of the CCM report on efforts to adapt the ACIC to the specific practice organization or even created a new one with special emphasis on specific items from the original version whereas others were not assessed [6,7]. However we reached the decision to develop a German translation addressing all six areas of the original ACIC and not to develop a new instrument.

Overall the ACIC subscale scores obtained in the Swiss samples were lower than the original scores of Bonomi et al. [2]. Empirically institutions begin with average scores below "5" on some or all areas of the ACIC. Comparison between the three different Swiss primary care settings showed higher scores in managed care practices, which were nearly comparable to the original scores. The higher scores in the managed care practices are likely to reflect the "culture" of these organizations. For example the mediX organization has been one of the first managed care organizations in Switzerland focusing on gate keeping and on a team based patient centred approach in health care. Electronic health records exist and the organization is active regarding quality improvement and caring for people with chronic illnesses. Particularly for the chosen condition diabetes strategies for coordinated care are available [8]. Most GPs in Switzerland however practice like in our study population solo or in small group organizations with one or two medical assistants. Their relationship is often more hierarchical than team based. Lack of resources and the still predominant tradition of paternalism instead of partnership and multiprofessional collaborative care can explain the low scores in delivery system design and community linkages. A survey in Germany with primary care physicians either working solo or in an organization in cities with 20'000 to 1'000'000 inhabitants showed that barriers and difficulties regarding community linkages were mentioned due to time constraints but also lacking motivation by the patients [9]. The low scores for information systems are not surprising taking into account that the majority of the small practices in Switzerland lack electronic health records, registries and reminder systems.

Implementation of self-management support as a central element of the CCM [1,10] and a key component in diabetes care was low in all three settings. Our own data for asthma primary care in Switzerland showed that the majority of patients get information only but not the skills necessary for self-management [11]. Reported reasons include the lack of confidence of health professionals in patients self-managing their own condition, dislike of self-management because misinterpretation as being disempowered, fearing loss of income, lack of time [12] and inadequate training in teaching patients self-management skills [13]. Finally the partnership and health care quality paradigm within the CCM concept is not supported by the payment system in Switzerland, a major barrier which is also known in other countries [1,14].

The organizations for the initial testing of the German version of the ACIC in Switzerland may not be representative for all health care organizations in other German speaking countries. However it can be assumed that the difficulties in transforming usual care into care congruent with the Chronic Care Model are similar.

Future projects should evaluate the German ACIC in different health care settings in German speaking countries. Relationships between the quality of chronic care delivered by the institution (ACIC) and the patients own view assessed by the validated German version of the patient assessment of chronic Illness care (PACIC) should be answered by further research [15-17].

## Conclusion

Clinicians and researchers benefit from a tool in their own language to assess whether health care is in alignment with the Chronic Care Model. The German version of the ACIC takes a step forward on the journey to best practice for chronic illness care in German speaking countries.

## Competing interests

The authors declare that they have no competing interests

## Authors' contributions

CSS and TR were the initiators and devised the conceptual framework of the paper. CSS drafted the report which the paper is based on and wrote the paper but all authors contributed in writing and revising the manuscript. AF did the statistical analysis.

CSS and AF translated the original version into German, SMK did the back translation into English and GSM was the leader of the group for the comprehensibility testing. All authors participated in consensus meetings and discussions.

All authors read and approved the final manuscript.

## Supplementary Material

Additional file 1 **The German assessment of Chronic Illness Care: G-ACIC**. The German version consist of 28 items covering the six areas of the Chronic Care Model plus in congruence with the actual version 3.5 of the ACIC six additional items that address how well a practice team or organization integrates the Chronic Care Model elements. Click here for file
