# Supplementary material for: Assessment of Chronic Illness Care with the German version of the ACIC in different primary care settings in Switzerland
Source: Health Qual Life Outcomes. 2010 Oct 27;8:122. doi: 10.1186/1477-7525-8-122 (PMC2988060; doi:10.1186/1477-7525-8-122)
Supplement: Additional file 1 — The German assessment of Chronic Illness Care: G-ACIC. The German version consist of 28 items covering the six areas of the Chronic Care Model plus in congruence with the actual version 3.5 of the ACIC six additional items that address how well a practice team or organization integrates the Chronic Care Model elements. [file 1477-7525-8-122-S1.PDF]

Erhebung der Versorgungsprozesse bei chronisch Kranken
German Assessment of Chronic Illness Care (G-ACIC version 3.5)

Bitte füllen Sie den folgenden Fragebogen über Ihre Institution aus. Die Informationen werden vertraulich behandelt. Bitte geben Sie Ihre Telefonnummer und E-Mail-Adresse an für den Fall, dass es für uns nötig ist, Sie oder Ihr Team in Zukunft zu kontaktieren. Bitte geben Sie auch die Namen der Personen an (z.B. Teammitglieder), die den Fragebogen ausfüllen.

**Ihr Name:**	**Datum:** ________/________/________<br>Tag Monat Jahr
**Institution und Adresse:**	**Namen von anderen Personen, die zusammen mit Ihnen den Fragebogen ausfüllten:**
	1.
	2.
	3.
**Ihre Telefonnummer:** (______) __ __ __ - __ __ __ __	**Ihre E-Mail-Adresse:**

Anweisung zum Ausfüllen des Fragebogen

Dieser Fragebogen wurde entwickelt, um Systeme und Versorger dabei zu unterstützen, den aktuellsten Wissensstand bei der Betreuung chronisch Kranker umzusetzen. Die Resultate können Ihnen und Ihrem Team helfen, Bereiche mit Verbesserungspotential zu erkennen.

1. **Bitte beantworten Sie jede Frage** aus der Perspektive Ihres Arbeitsumfeldes (z.B. Praxis, Klinik, Spital, andere Gesundheitsinstitutionen), die die Versorgung chronisch Kranker unterstützt.

   Bitte geben Sie Namen und den Typ der Institution an (z.B. Einzelpraxis, Ärztenetzwerk, Poliklinik, Gruppenpraxis)

   ____

2. **Beantworten Sie jede Frage** indem Sie berücksichtigen, wie Ihre Institution aktuell im Hinblick auf chronische Erkrankungen verfährt.

   Bitte spezifizieren Sie die Krankheit/das Gesundheitsproblem____________________________

3. **Bitte kreisen Sie** in jeder Reihe **denjenigen Wert ein**, der am Besten die Versorgung beschreibt, die momentan in Ihrer Institution für die genannte chronische Krankheit stattfindet. Die Reihen beschreiben die Schlüsselaspekte der Versorgung von chronisch Kranken. Die verschiedenen Stufen jedes Aspektes beschreiben Qualitätsebenen der Versorgung. Die Stufen sind durch Werte zwischen 0 bis 11 dargestellt, 0 zeigt die tiefste und 11 die höchste Umsetzung an.

Für mehr Informationen über das Ausfüllen des Fragebogens wenden Sie sich bitte an:
Institut für Hausarztmedizin der Universität Zürich,
Sonneggstrasse 6,
8091 Zürich, Switzerland
www.hausarztmedzin.uzh.ch

Copyright 2000, The MacColl Institute for Healthcare Innovation, Group Health Cooperative
German version Assessment of Chronic Illness Care; G-ACIC; University of Zurich

Erhebung der Versorgungsprozesse bei chronisch Kranken, G-ACIC Version 3.5

Teil 1: Organisation Ihrer Institution (Gesundheitsversorgungssystem)

Komponenten	Level D	Level C	Level B	Level A
**Eine übergreifende organisatorische Führung in der Versorgung von chronischen Krankheiten**	…existiert nicht oder es ist ein geringes Interesse vorhanden.	…zeigt sich in Form von Visionen und Businessplänen, aber es werden keine Ressourcen dafür bereit gestellt.	…zeigt sich in Führung und zur Verfügung gestellten Ressourcen (Geld und Personal).	…ist ein Teil der Langzeitstrategie, nötige Ressourcen sind vorhanden und spezifische Personen für die Umsetzung verantwortlich.
**Score**	0 1 2	3 4 5	6 7 8	9 10 11
**Organisationsziele für die Versorgung von chronisch Kranken**	…existieren nicht oder sind auf eine Krankheit beschränkt.	…existieren aber werden nicht aktiv überprüft.	…sind messbar und werden überprüft.	…sind messbar, werden routinemässig überprüft und sind in Verbesserungsplänen enthalten.
**Score**	0 1 2	3 4 5	6 7 8	9 10 11
**Verbesserungsstrategien für die Versorgung chronisch Kranker**	… sind ad hoc und nicht konsistent organisiert oder abgestützt.	…verwenden ad hoc Ansätze für bestimmte Probleme, wenn sie auftreten.	…verwenden eine bewährte Verbesserungsstrategie für bestimmte Probleme.	…verwenden eine bewährte Verbesserungsstrategie proaktiv, um Organisationsziele zu erreichen.
**Score**	0 1 2	3 4 5	6 7 8	9 10 11
**Ausführungsrichtlinien für die Versorgung chronisch Kranker**	…werden nicht verwendet, um die klinische Leistung zu beeinflussen.	…werden verwendet, um Nutzen und Kosten der Versorgung von Patienten mit chronischen Krankheiten zu beeinflussen.	…werden verwendet, um Patienten- Versorgungsziele zu unterstützen.	…werden verwendet, um Versorger zu motivieren und zu befähigen, Patienten-Versorgungsziele zu unterstützen.
**Score**	0 1 2	3 4 5	6 7 8	9 10 11
**Vorgesetzte**	… entmutigen bei Verbesserungsbestrebungen in der Versorgung chronisch Kranker.	…machen Verbesserungen bei der Versorgung von Patienten mit chronischen Krankheiten nicht zu einer Priorität.	…ermutigen zu Verbesserungen bei der Versorgung chronisch Kranker.	…nehmen sichtbar teil in den Verbesserungsbestrebungen in der Versorgung chronisch Kranker.
**Score**	0 1 2	3 4 5	6 7 8	9 10 11
**Zuschüsse**	…unterstützen das Selbstmanagement von Patienten oder Systemveränderungen nicht.	… weder unterstützen noch behindern sie das Selbstmanagement von Patienten, Systemveränderungen, oder beides.	... unterstützen das Selbstmanagement von Patienten oder Systemveränderungen.	...sind spezifisch gestaltet, um die Versorgung von Patienten mit chronischen Krankheiten zu verbessern.
**Score**	0 1 2	3 4 5	6 7 8	9 10 11

Copyright 2000, The MacColl Institute for Healthcare Innovation, Group Health Cooperative

Gesamtwert Teil 1: ________ Durchschnittswert Teil 1 (Gesamtwert / 6): _________

Copyright 2000, The MacColl Institute for Healthcare Innovation, Group Health Cooperative

Teil 2: Vernetzung mit Gemeinwesen / lokalen Ressourcen

Komponenten	Level D	Level C	Level B	Level A
**Vernetzung der Patienten mit lokalen Ressourcen**	...wird nicht systematisch gemacht.	…ist beschränkt auf eine zugängliche Liste mit bekannten lokalen - Ressourcen.	…wird durch einen bestimmten Mitarbeiter sichergestellt, damit eine optimale Nutzung lokaler Ressourcen erfolgt.	…wird durch aktive Koordination zwischen Gesundheitssystem, lokalen Angeboten und Patienten erreicht.
**Score**	0 1 2	3 4 5	6 7 8	9 10 11
**Kooperationen mit lokalen Institutionen**	...existieren nicht.	…werden in Betracht gezogen, aber bis jetzt nicht umgesetzt.	…wurden eingegangen, um unterstützende Programme und Vorgehensweisen zu entwickeln.	…werden aktiv gesucht, um systemübergreifende Programme und Strategien zu entwickeln.
**Score**	0 1 2	3 4 5	6 7 8	9 10 11
**Kantonale/nationale Gesundheitspläne**	…koordinieren Richtlinien zu chronischen Erkrankungen, Massnahmen oder Betreuungsressourcen auf Praxisebene nicht.	…ziehen in einen gewissen Grad Koordination von Richtlinien, Massnahmen oder Betreuungsressourcen in Erwägung, haben aber noch keine Veränderungen umgesetzt.	…koordinieren gegenwärtig Richtlinien, Massnahmen oder Betreuungsressourcen auf einem oder zwei Gebieten von chronischen Krankheiten.	…koordinieren gegenwärtig Richtlinien, Massnahmen oder Betreuungsressourcen für die meisten chronischen Krankheiten auf Praxisebene.
**Score**	0 1 2	3 4 5	6 7 8	9 10 11

Gesamtwert Teil 2: ________ Durchschnittswert Teil 2 (Gesamtwert / 3): _________

Copyright 2000, The MacColl Institute for Healthcare Innovation, Group Health Cooperative

Teil 3: Praxis Ebene

Teil 3a: Unterstützung des Selbstmanagements

Komponenten	Level D	Level C	Level B	Level A
**Erhebung und Dokumentation von Selbstmanagement Bedürfnissen und Aktivitäten**	...werden nicht gemacht.	...werden nicht systematisch erhoben.	…werden in einer standardisierten Art und Weise erhoben.	…werden regelmässig und standardisiert erhoben und mit einem für Praxis und Patienten verfügbaren Behandlungsplan gekoppelt.
**Score**	0 1 2	3 4 5	6 7 8	9 10 11
**Unterstützung des Selbstmanagements**	…ist beschränkt auf die Abgabe von Informationen (Infoblätter, Broschüren).	…ist verfügbar durch die Überweisung an Selbstmanagement Kursen oder Instruktoren/Edukatoren.	...wird von geschulten klinischen Fachpersonen angeboten mit Expertise in Selbstmanagementunterstützung; mit denen eine enge klinische Zusammenarbeit besteht.	…wird von spezifisch in Patientenbefähigung und Problemlösung geschulten klinischen Fachpersonen angeboten, die die meisten Patienten mit chronischen Krankheiten sehen und an die jeweilige Institution angegliedert sind.
**Score**	0 1 2	3 4 5	6 7 8	9 10 11
**Eingehen auf Anliegen von Patienten und Familien**	...geschieht nicht systematisch.	...wird für spezifische Patienten- und Angehörigenanliegen durch Überweisung ermöglicht	...wird gefördert, und Unterstützung durch Selbsthilfegruppen und Betreuungsprogramme ist vorhanden.	...ist ein integraler Bestandteil der Versorgung und beinhaltet systematische Erhebungen und routinemässige Einbindung in Unterstützungsprogramme.
**Score**	0 1 2	3 4 5	6 7 8	9 10 11
**Effektive Interventionen zur Verhaltensänderung und Unterstützung durch andere Betroffene (peer support)**	...sind nicht verfügbar.	…sind beschränkt auf die Abgabe von Infoblättern, Broschüren oder andere schriftliche Informationen.	…sind nur auf Überweisung an spezialisierte Zentren mit geschultem Personal verfügbar.	…sind leicht verfügbar und ein integraler Bestandteil der routinemässigen Versorgung.
**Score**	0 1 2	3 4 5	6 7 8	9 10 11

Gesamtwert Teil 3a: ________ Durchschnittswert Teil 3a (Gesamtwert / 4): _________

Copyright 2000, The MacColl Institute for Healthcare Innovation, Group Health Cooperative

Teil 3b: Entscheidungsunterstützung

Komponenten	Level D	Level C	Level B	Level A
**Evidenzbasierte Richtlinien (Guidelines)**	...sind nicht verfügbar.	...sind verfügbar aber nicht in das Versorgungsangebot integriert.	...sind verfügbar und werden durch Schulung der Anbieter berücksichtigt.	...sind verfügbar, werden durch Schulung der Anbieter und durch Remindersysteme und andere bewährte Methoden zur Verhaltensänderung in die Versorgung integriert.
**Score**	0 1 2	3 4 5	6 7 8	9 10 11
**Involvierung von Spezialisten zur Verbesserung der Grundversorgung**	…geschieht hauptsächlich durch traditionelle Überweisung.	… wird erzielt und die Kapazität des Systems für die Implementierung von Richtlinien in die Routineversorgung wird durch die Spezialisten ausgebaut.	…beinhaltet die Anleitung und Weiterbildung des Grundversorger-Teams durch ausgewiesene Spezialisten.	…beinhaltet die Involvierung und Anleitung durch Spezialisten zur Verbesserung der Betreuung der Patienten in der Grundversorgung.
**Score**	0 1 2	3 4 5	6 7 8	9 10 11
**Schulung der Anbieter im Management chronischer Krankheiten**	...wird sporadisch angeboten.	…wird systematisch durch traditionelle Methoden (z.B. Wissensvermittlung durch Frontalunterricht) angeboten.	…wird durch den Einsatz von validierten Methoden (z. B. basierend auf Lerntheorien, Verhaltensmodellen) angeboten.	...beinhaltet das Training aller Praxisteams in den Methoden zur Versorgung chronischer Krankheiten wie populations-basiertes Management und Selbst-Management-Unterstützung.
**Score**	0 1 2	3 4 5	6 7 8	9 10 11
**Patienteninformation über die Richtlinien (Guidelines)**	... wird nicht gemacht.	…kann vom Patienten angefordert oder abgerufen werden.	...die spezifischen Patientenschulungs-Materialien werden den Patienten für jede Richtlinie abgegeben.	...beinhaltet die Entwicklung von spezifischen Materialen für Patienten, die ihre Rolle in der Einhaltung (Adhärenz mit) der Richtlinie beschreiben.
**Score**	0 1 2	3 4 5	6 7 8	9 10 11

Gesamtwert Teil 3b: ________ Durchschnittswert Teil 3b (Gesamtwert / 4): _________

Copyright 2000, The MacColl Institute for Healthcare Innovation, Group Health Cooperative

Teil 3c: Der Aufbau des Versorgungssystems

Komponenten	Level D	Level C	Level B	Level A
**Die Arbeitsweise des Praxisteams**	…wird nicht berücksichtigt.	…wird berücksichtigt, indem die Verfügbarkeit von Personen mit angemessenem Training in chronischem Krankheitsmanagement sicher gestellt wird.	...wird gewährleistet durch regelmässige Teamsitzungen, in denen Richtlinien, Rollen und Verantwortlichkeiten sowie Probleme bei der Versorgung von chronisch Kranken behandelt werden.	...wird gewährleistet durch regelmässige Teamtreffen, mit klar definierter Rollenverteilung einschliesslich Selbst-Management Schulung, proaktive Nachkontrolle, Koordination der Ressourcen.
**Score**	0 1 2	3 4 5	6 7 8	9 10 11
**Führung des Praxisteams**	…ist nicht erkennbar.	…wird für spezifische organisatorische Aufgaben angenommen.	…wird durch die Benennung eines Teamleaders gewährleistet, dessen Rolle im Management chronischer Krankheiten aber nicht definiert ist.	...wird durch die Benennung eines Teamleaders garantiert, der gewährleistet, dass Aufgaben und Verantwortlichkeit für die Betreuung chronisch Kranker klar definiert sind.
**Score**	0 1 2	3 4 5	6 7 8	9 10 11
**Terminsystem**	…kann zur Planung von Akutversorgung sowie Folge- und präventiv ausgerichtete Konsultationen verwendet werden.	...stellt Nachkontrolltermine für chronisch kranke Patienten sicher.	...ist flexibel und kann Innovationen wie individuelle Dauer der Termine oder Gruppenkonsultationen aufnehmen.	...erleichtert die Organisation von Konsultation bei mehreren Versorgern in einer einzelnen Visite.
**Score**	0 1 2	3 4 5	6 7 8	9 10 11
**Folgevisiten/ Nachkontrollen**	…werden durch Patienten oder Versorger „ad hoc“ vereinbart.	…werden durch die Praxis in Übereinstimmung mit den Richtlinien festgelegt.	...die Einhaltung der Folgevisiten wird durch das Praxisteam überprüft und gewährleistet.	…werden auf die Bedürfnisse der Patienten ausgerichtet, variieren in Intensität und Methode (telefonisch, persönlich, über E-Mail) und erfolgen gemäss den Richtlinien.
**Score**	0 1 2	3 4 5	6 7 8	9 10 11
**Spezifische Visiten für chronische Krankheitsversorgung**	...werden nicht angeboten oder sind nicht verfügbar.	...werden gelegentlich für komplizierte Patienten angeboten.	...sind ein vorhandenes Angebot für interessierte Patienten.	...werden allen chronisch Kranken angeboten und schliessen regelmässige Kontrollen/Erhebungen, präventive Interventionen und ein Augenmerk auf Unterstützung des Selbstmanagements mit ein.
**Score**	0 1 2	3 4 5	6 7 8	9 10 11

Copyright 2000, The MacColl Institute for Healthcare Innovation, Group Health Cooperative

**Komponenten**	**Level D**	**Level C**	**Level B**	**Level A**
**Kontinuität der Versorgung**	…findet keine prioritäre Berücksichtigung.	…ist abhängig von der schriftlichen Kommunikation zwischen den Grundversorgern und den Spezialisten, Case-Managern.	... zwischen den Grundversorgern und Spezialisten oder andern Anbietern ist eine Priorität, wird aber nicht systematisch berücksichtigt.	…hat eine hohe Priorität und alle Interventionen bei chronischen Krankheiten beinhalten eine aktive Koordination zwischen Grundversorgern, Spezialisten und anderen relevanten Gruppen.
**Score**	0 1 2	3 4 5	6 7 8	9 10 11

Gesamtwert Teil 3c: ________ Durchschnittswert Teil 3c (Gesamtwert / 6): _________

Copyright 2000, The MacColl Institute for Healthcare Innovation, Group Health Cooperative

Teil 3d: Klinische Informationssysteme

Komponenten	Level D	Level C	Level B	Level A
**Patientenregister/ Datenbank (Erfassung aller Patienten mit spezifischen chronischen Erkrankung)**	...existiert nicht.	…beinhaltet Namen, Diagnose, Kontaktinformationen und Datum der letzten Konsultation, entweder in schriftlicher oder elektronischer Form.	…hat eine Suchfunktion, um Subpopulationen nach klinisch-medizinischer Wertigkeit zu ermitteln.	...ist an Richtlinien geknüpft, welche an vorzunehmende Untersuchungen erinnern.
**Score**	0 1 2	3 4 5	6 7 8	9 10 11
**Remindersysteme für Betreuende/Versorger**	...existieren nicht.	…weisen auf das Vorliegen einer chronischen Krankheit hin, beschreiben aber die erforderlichen Untersuchungen nicht.	…weisen auf notwendige Untersuchungen durch regelmässige Rückmeldung hin.	... beinhalten spezifische Informationen für das Team zur Einhaltung der Richtlinien bei der Patienten Konsultation.
**Score**	0 1 2	3 4 5	6 7 8	9 10 11
**Feedback**	…wird nicht gegeben oder ist nicht Team spezifisch.	...wird selten und nicht persönlich gegeben.	...erfolgt in regelmässigen Zeitabständen zur Überprüfung der Leistung und ist spezifisch für das Team.	...erfolgt regelmässig, Team spezifisch und wird routinemässig persönlich durch einen akzeptierten Meinungsträger gegeben, um die Teamleistung zu verbessern.
**Score**	0 1 2	3 4 5	6 7 8	9 10 11
**IT-Informationen über unterstützungsbedürftige Patienten**	...sind nicht verfügbar.	...können nur mit besonderem Aufwand oder zusätzlicher Programmierung eingeholt werden.	...können auf Anfrage eingeholt werden sind aber nicht routinemässig verfügbar.	...werden den Versorgern routinemässig bereitgestellt, um ihnen zu helfen, die vorgesehene Versorgung zu gewährleisten.
**Score**	0 1 2	3 4 5	6 7 8	9 10 11
**Patienten-Behandlungspläne**	...werden nicht erstellt.	… werden standardisiert erstellt.	…werden von allen Beteiligten zusammen erstellt und beinhalten sowohl Selbstmanagementziele als auch klinische Ziele.	…werden von allen Beteiligten zusammen erstellt und beinhalten Selbstmanagement sowie klinisches Management. Verlaufskontrollen finden statt und leiten die weitere Betreuung.
**Score**	0 1 2	3 4 5	6 7 8	9 10 11

Gesamtwert Teil 3d: ________ Durchschnittswert Teil 3d (Gesamtwert / 5): _________

Copyright 2000, The MacColl Institute for Healthcare Innovation, Group Health Cooperative

Integration der Elemente des Chronic Care Models

Komponenten	Wenig Unterstützung	Einfache Unterstützung	Gute Unterstützung	Volle Unterstützung
**Patienteninformation gemäss Richtlinien (Guidelines)**	...erfolgt nicht.	…erfolgt auf Anfrage oder über das Anbieter eigene System.	…erfolgt mittels spezifischer Patientenschulungsmaterialen gemäss Richtlinien.	…beinhaltet spezifische, für Patienten entwickelte Materialien, die ihre Rolle im Umsetzen der Richtlinienziele beschreiben.
**Score**	0 1 2	3 4 5	6 7 8	9 10 11
**Informationssysteme / Patientenregister/-datenbanken**	…beinhalten keine Selbstmanagementziele von Patienten.	…beinhalten Resultate von Patientenerhebungen (z.B. Einschätzung des Funktionszustandes, Bereitschaft, an Selbst-Managementaktivitäten teilzunehmen) aber keine Ziele.	...beinhalten sowohl Resultate von Patientenuntersuchungen als auch Selbstmanagementziele, die unter Mitsprache von Praxisteam, Versorgern und Patienten entwickelt wurden.	...beinhalten sowohl Resultate von Patientenuntersuchungen als auch Selbstmanagementziele, die unter Beteiligung von Praxisteam, Versorgern und Patienten entwickelt wurden sowie Reminder für Patienten und/oder Versorger zur Nachkontrolle und regelmässige Zielüberprüfung.
**Score**	0 1 2	3 4 5	6 7 8	9 10 11
**Lokale Programme des Gemeinwesens (z.B. Ligen, Spitex, ProSenectute..)**	… geben der Institution (Praxis / Klinik etc.) keinen Feedback über den Fortschritt der Patienten in ihren Programmen.	… geben sporadisches Feedback an gemeinsamen Sitzungen zwischen den Anbietern und der Institution (Praxis / Klinik etc.) über den Fortschritt der Patienten in ihren Programmen.	... geben der Institution (Praxis / Klinik etc.) regelmässiges Feedback durch formale Zwischenberichte über den Fortschritt der Patienten.	... geben der Institution (Praxis / Klinik etc.) regelmässiges Feedback über den Fortschritt jedes einzelnen Patienten unter Mitsprache des Patienten, um die Programme gemäss den Patientenbedürfnissen zu modifizieren.
**Score**	0 1 2	3 4 5	6 7 8	9 10 11

Copyright 2000, The MacColl Institute for Healthcare Innovation, Group Health Cooperative

Komponenten	Wenig Unterstützung	Einfache Unterstützung	Gute Unterstützung	Volle Unterstützung
**Die Planung der Institution** (Praxis / Klinik etc.) **für die Betreuung chronisch Kranker**	...schliesst keinen auf die Gesamtbevölkerung basierten Ansatz ein.	...verwendet Daten von Informationssystemen, um die Versorgung zu planen.	...verwendet Daten von Informationssystemen, um eine populationsbasierte Versorgung proaktiv zu planen einschliesslich der Entwicklung von Selbst-Managementprogrammen und Partnerschaften mit lokalen Ressourcen.	...verwendet systematisch Daten und Input des Praxisteams, um die Versorgung proaktiv zu planen einschliesslich der Entwicklung von Selbstmanagement Programmen und Partnerschaften mit lokalen Anbietern. Die Planung beinhaltet einen Evaluationsplan, um den Erfolg über die Zeit zu bestimmen.
**Score**	0 1 2	3 4 5	6 7 8	9 10 11
**Routinemässige Folgevisiten für Arzttermine, strukturierte Patientenuntersuchungen und Zielplanung**	...sind nicht sichergestellt.	...werden sporadisch gemacht, normalerweise nur für Terminvereinbarungen.	...sind sichergestellt, indem Verantwortung an spezifisches Personal (z.B.MPA, Case-Manager) übertragen wird.	...sind sichergestellt, indem Verantwortung an spezifisches Personal (z.B. MPA, Case-Manager) übertragen wird. Dieses nutzt Patientenregister und andere Remindersysteme zur Koordination zwischen Patienten und dem gesamten Praxisteam.
**Score**	0 1 2	3 4 5	6 7 8	9 10 11
**Richtlinien für die Versorgung von Patienten mit chronischen Krankheiten**	...werden Patienten nicht zur Verfügung gestellt.	...werden denjenigen Patienten zur Verfügung gestellt, die ausdrücklich am Selbstmanagement ihrer Krankheit interessiert sind.	...werden allen Patienten zur Verfügung gestellt mit dem Ziel, sie beim Aufbau von wirksamem Selbstmanagement, Verhalten oder Verhaltensänderungen zu unterstützen sowie die Erfordernis zur rechtzeitige Kontaktaufnahme mit dem Praxisteam zu erkennen.	...werden durch das Praxisteam mit den Patienten überprüft, mit dem Ziel ein Selbstmanagement- oder Verhaltensänderungs-Programm festzulegen, das mit den Richtlinien in Einklang ist und die Ziele des Patienten und dessen Bereitschaft zu Veränderung berücksichtigt.
**Score**	0 1 2	3 4 5	6 7 8	9 10 11

Gesamtwert Integration ________ Durchschnittswert Integration (Gesamtwert / 6): _________

Copyright 2000, The MacColl Institute for Healthcare Innovation, Group Health Cooperative

Bitte beschreiben Sie kurz, wie Sie das Formular ausfüllten (z.B. erzielte Übereinstimmung in einer persönlichen Sitzung, ausgefüllt vom Teamleader/Gruppenleiter unter Einbezug von anderen Teammitgliedern wenn erforderlich, jedes Gruppenmitglied füllte ein Formular einzeln aus und die Antworten wurden gemittelt).

Beschreibung: ____

____

____

Übertragung der Gesamtwerte der einzelnen Abschnitte

Gesamtwert Teil 1: Organisation Ihrer Institution (Gesundheitsversorgungssystem) _______

Gesamtwert Teil 2: Vernetzung mit Gemeinwesen / lokalen Ressourcen _______

Gesamtwert Teil 3a: Unterstützung des Selbstmanagements _______

Gesamtwert Teil 3b: Entscheidungsunterstützung _______

Gesamtwert Teil 3c: Der Aufbau des Versorgungssystems _______

Gesamtwert Teil 3d: Klinische Informationssysteme (IT) _______

Gesamtwert Teil Integration: Integration der Elemente des Chronic Care Models _______

Summe aller Gesamtwerte _______

Durchschnittswert (Summe aller Gesamtwerte /7) _______

Copyright 2000, The MacColl Institute for Healthcare Innovation, Group Health Cooperative

Was bedeutet die Punkteauswertung?

Der G-ACIC ist so aufgebaut, dass der höchste Wert (11) bei einer einzelnen Frage, einer Subskala oder dem Gesamtwert (Durchschnittswert der sechs ACIC Subskalen) einem optimalen Betreuungsangebot und der kleinst mögliche Wert (0) einem limitierten Betreuungsangebot für Patienten mit chronischen Krankheiten entspricht. Die Richtlinien für die Interpretation sind wie folgt:

Zwischen 0 und 2:	Limitiertes Betreuungsangebot für Patienten mit chronischen Krankheiten
Zwischen 3 und 5:	Basis-Betreuungsangebot für Patienten mit chronischen Krankheiten
Zwischen 6 und 8:	Ziemlich gutes Betreuungsangebot für Patienten mit chronischem Krankheiten
Zwischen 9 und 11:	Voll entwickeltes Betreuungsangebot für Patienten mit chronischen Krankheiten

Es ist relativ typisch, zu Beginn Durchschnittswerte von unter 5 in einigen (oder allen) Bereichen des G-ACIC zu erzielen.
Schliesslich würde kein Bedarf zur Qualitätsverbesserung bestehen, wenn eine optimale Versorgung für chronisch Kranke bereits gegeben wäre.
Es ist auch üblich, dass Versorger zu Beginn eine bessere Versorgung von Patienten mit chronischen Erkrankungen annehmen, als dies tatsächlich der Fall ist.
Im Laufe der Zeit werden Sie und Ihr Team vertrauter damit werden, was ein effektives Versorgungssystem alles umfasst. Es ist sogar möglich, dass die Werte vorübergehend abnehmen, selbst wenn Fortschritte erzielt werden. Dies ist meistens das Resultat des besseren Verständnisses. Mit der Zeit wird eine Verbesserung der Gesamtwerte beobachtet.

Copyright 2000, The MacColl Institute for Healthcare Innovation, Group Health Cooperative
